# Characterizing RNA ensembles from NMR data with kinematic models

**DOI:** 10.1093/nar/gku707

**Published:** 2014-08-11

**Authors:** Rasmus Fonseca, Dimitar V. Pachov, Julie Bernauer, Henry van den Bedem

**Affiliations:** 1 AMIB Project, INRIA Saclay-Île de France, 1 rue Honoré d'Estienne d'Orves, Bâtiment Alan Turing, Campus de l’École Polytechnique, 91120 Palaiseau, France; 2 Laboratoire d'Informatique de l’École Polytechnique (LIX), CNRS UMR 7161, École Polytechnique, 91128 Palaiseau, France; 3 Department of Computer Science, University of Copenhagen, Nørre Campus, Universitetsparken 5, DK-2100 Copenhagen, Denmark; 4 Department of Chemistry, Stanford University, 333 Campus Dr., Stanford, CA 94305, USA; 5 Joint Center for Structural Genomics, Stanford Synchrotron Radiation Lightsource, Stanford University, 2575 Sand Hill Road, Menlo Park, CA 94025, USA

## Abstract

Functional mechanisms of biomolecules often manifest themselves precisely in transient conformational substates. Researchers have long sought to structurally characterize dynamic processes in non-coding RNA, combining experimental data with computer algorithms. However, adequate exploration of conformational space for these highly dynamic molecules, starting from static crystal structures, remains challenging. Here, we report a new conformational sampling procedure, KGSrna, which can efficiently probe the native ensemble of RNA molecules in solution. We found that KGSrna ensembles accurately represent the conformational landscapes of 3D RNA encoded by NMR proton chemical shifts. KGSrna resolves motionally averaged NMR data into structural contributions; when coupled with residual dipolar coupling data, a KGSrna ensemble revealed a previously uncharacterized transient excited state of the HIV-1 trans-activation response element stem–loop. Ensemble-based interpretations of averaged data can aid in formulating and testing dynamic, motion-based hypotheses of functional mechanisms in RNAs with broad implications for RNA engineering and therapeutic intervention.

## INTRODUCTION

Non-coding ribonucleic acids (ncRNAs) mediate important cellular processes. Transfer RNA and ribosomal RNA are essential functional components in protein synthesis (1). Short interfering RNAs (siRNAs) and microRNAs (miRNAs) are the effector molecules in RNA interference, the process of silencing expression of specific genes in cells, and hold great promise as therapeutics (2,3). Riboswitches regulate gene expression by adopting alternative, 3D conformations in response to binding events (4). In RNA nanomedicine, these and other functional RNAs are fused into nanoparticles for targeted intracellular delivery, silencing cancer and infectious disease-specific genes (5).

RNA molecules are highly dynamic, sampling a wide range of conformational rearrangements to interact with binding partners and perform their function (6,7). The native ensemble of biomolecules, i.e. the set conformational states the molecule adopts *in vivo*, cannot be observed directly. Solution-state nuclear magnetic resonance (NMR) spectroscopy can probe the RNA conformational landscape at timescales ranging from picosecond to seconds or longer, often providing detailed evidence of dynamically interchanging, sparsely populated substates (8,9). Structurally characterizing conformational substates would offer tremendous potential for uncovering functional mechanisms (10), particularly for riboswitches (11), or predicting molecular interactions of RNA sub-units, such as in nanostructures (12). However, resolving motionally averaged NMR measurements into constituent, structural contributions that represent key features of the data remains extremely challenging (13).

**Figure 1.**
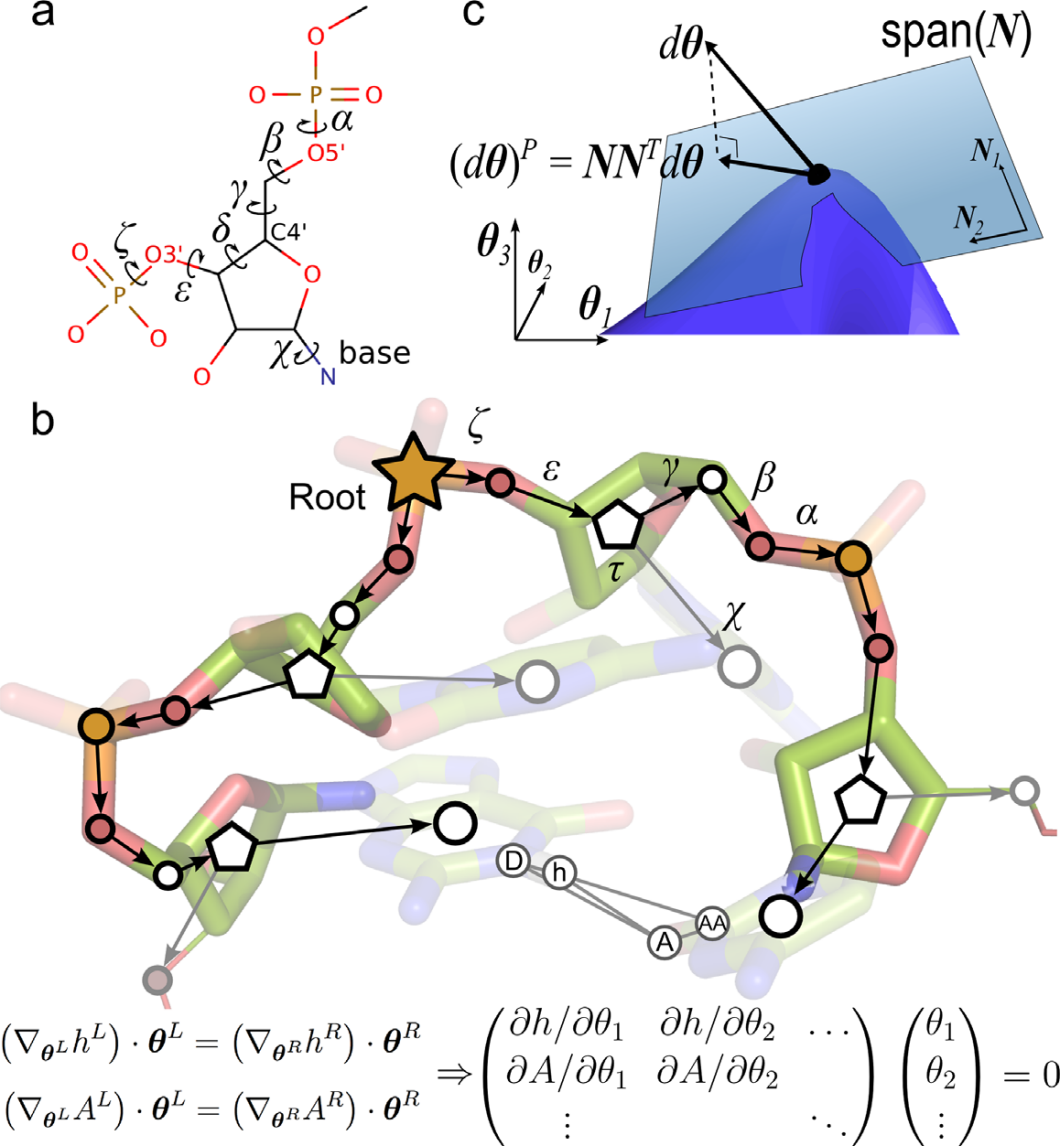
Kinematic representation of RNA. (**a**) A single nucleotide of RNA with its torsional degrees-of-freedom. (**b**) Edges in the directed spanning tree encode *n* torsional degrees-of-freedom $\boldsymbol{\theta } = (\theta _1, \ldots , \theta _n)$ and vertices (circles) encode rigid bodies. Pentagons represent riboses, which have an additional internal degree-of-freedom governing their conformation (puckering). The hydrogen bond *h*-A closes a kinematic cycle, and is one of *m* distance constraints. As the position of the hydrogen atom *h* changes through perturbation of dihedral angles in the left branch of the tree, the new position of *h* should be matched by appropriate changes in the right branch, i.e. $\left(\nabla _{{\boldsymbol{\theta }}^{\rm L}} h^{\rm L}\right)\cdot {\boldsymbol{\theta }}^{\rm L} = \left(\nabla _{{\boldsymbol{\theta }}^{\rm R}} h^{\rm R}\right)\cdot {\boldsymbol{\theta }}^{\rm R}$. Similarly, a change in position of heavy atom A from the right tree should be matched by changes in the left tree. These instantaneous distance constraints define the 6*m* × *n* Jacobian matrix $\boldsymbol{J}$. (**c**) A schematic representation of the subspace of conformational space defined by the closure constraints. The subspace (blue surface) is highly nonlinear, but can be locally approximated by its tangent space, the null-space of $\boldsymbol{J}$ (translucent blue plane).

The value of analyzing NMR spectroscopy data guided by a conformational ensemble has long been recognized (14,15). Conformational diversity for RNA ensemble analyses is often provided by sophisticated molecular dynamics simulations (16,17). Long trajectories with specialized force fields on dedicated supercomputers are required to adequately sample conformational space, limiting ensemble analyses to modestly-sized RNA molecules (18). Here, we present an efficient conformational sampling procedure, Kino-geometric sampling for RNA (KGSrna), which can report on ensembles of RNA molecular conformations orders of magnitude faster than MD simulations. KGSrna represents an RNA molecule with rotatable, single bonds as degrees-of-freedom and groups of atoms as rigid bodies (Figure 1a). In this representation, non-covalent bonds form distance constraints, which create nested, closed rings (Figure 1b). Torsional degrees-of-freedom in a closed ring demand carefully coordinated changes to avoid breaking the non-covalent bond, which greatly reduces the conformational flexibility (19–22). The reduced flexibility from a network of nested, closed rings consequently deforms the biomolecule along preferred directions on the conformational landscape. In contrast to techniques based on explicit constraint counting (19,22), our new procedure projects degrees-of-freedom onto a lower-dimensional subspace of conformation space, in which the geometries of the non-covalent bonds are maintained exactly under conformational perturbation.

The dimensionality reduction additionally enables efficient exploration of conformational space and reduces the risk of overfitting sparse experimental data. Kino-geometric sampling of 3D RNA models can recover the conformational landscape encoded by proton chemical shifts in solution. Combined with NMR residual dipolar coupling (RDC) measurements, our procedure can automatically determine the size and weights of a parsimonious conformational ensemble that, provably, best agrees with the data. Our results can guide interpretation of proton chemical shift (23) or RDC data (16,17), and complement insights obtained from single, averaged models (24–26), ensembles resulting from experimentally guided modeling procedures (27–29), normal mode analysis (30), Monte Carlo simulations (31) or *de novo* tertiary structure prediction (32–34).

## MATERIALS AND METHODS

### A kinematic model for non-coding RNA

We encode a polynucleotide as a rooted, directed spanning tree, i.e. an acyclic graph that connects all vertices such that each one, except the root, has only one incoming, directed edge. Each vertex represents a rigid group of atoms (rigid body), and each edge represents a rotational (torsional) degree-of-freedom for the backbone (*α*, *β*, *γ*, *δ*, *ε* and *ζ*) or the N-glycosidic bond of the nucleoside (*χ*) (Figure 1a). We use the linear, branched structure of the polynucleotide to identify rigid bodies based only on knowledge of bond-flexibility. Initially each atom is a rigid body. Atoms that are (partially) double bonded have their rigid bodies merged and atoms with only one covalent neighbor are merged with their neighbor. Thus, rigid bodies are the largest conformational sub-units not containing internal rotational degrees-of-freedom.

Perturbing the torsional angle *δ* generally breaks the geometry of riboses. To efficiently sample ribose conformations we introduced a differentiable coordinate transformation *τ* for the angle *δ*, which maintains ideal geometry of the ribose when *δ* is perturbed (Supplementary Figure S1).

A vector ${\boldsymbol{\theta }} \in \mathbb {S}^n$, with $\mathbb {S}$ the unit circle, completely specifies a conformation for a molecule with *n* rotational degrees-of-freedom. Each hydrogen bond defines a closed ring or *kinematic cycle*. In this study, we consider hydrogen bonds between Watson−Crick (WC) pairs only. For any hydrogen bond, rotation along the *h*-A axis is allowed but no other distortion of the geometry is permitted (Figure 1b). Inter-atomic forces are implicitly encoded by rigid links between adjacent atoms and a long-range hard-sphere interaction potential based on van der Waals radii.

### Conformational perturbation with constraints

Randomly perturbing a conformation would break the hydrogen bonds. We developed two complementary conformational sampling mechanisms that, in linear approximation, maintain distance constraints exactly. A *null-space* perturbation sensitively samples local neighborhoods of conformation space and a *rebuild* perturbation can rapidly explore more distant areas (20).

#### Null-space perturbation

A null-space perturbation of a conformation $\boldsymbol{\theta }$ projects an *n*-dimensional trial-vector onto the null-space of the Jacobian matrix ${\boldsymbol{J}}$. This 6*m* × *n* matrix is defined by the instantaneous, or velocity, kinematic relation ${d \boldsymbol{x}} = {\boldsymbol{J}}d{\boldsymbol{\theta }}$, where $\boldsymbol{x}$ are the *m* 6D coordinates describing the end-effectors, i.e. the position and orientation of the donor and acceptor atoms that define *m* closure constraints (20,22,35) (Figure 1b). The matrix ***J*** can be obtained as follows. If $h^{{\rm L},{\rm R}}: \boldsymbol{\theta }^{{\rm L},{\rm R}} \mapsto (h_x, h_y, h_z, h_{\alpha }, h_{\beta }, h_{\gamma })$ is the map relating the degrees-of-freedom to the position and orientation of the hydrogen donor atom going around the left (L) or right (R) side of the cycle, then the constraint equations are $\left(\nabla _{{\boldsymbol{\theta }}^{\rm L}} h^{\rm L}\right)\cdot {\boldsymbol{\theta }}^{\rm L} = \left(\nabla _{{\boldsymbol{\theta }}^{\rm R}} h^{\rm R}\right)\cdot {\boldsymbol{\theta }}^{\rm R}$, i.e. infinitesimal displacements for *h* resulting from conformational changes from the left side of the cycle should match those from the right. Similar expressions hold for the acceptor atom A. This leads to six constraints per cycle. Thus, the entries of ${\boldsymbol{J}}$ contain the derivative of all hydrogen bond end-atom positions with respect to each degree-of-freedom (Figure 1b). The null-space of ${\boldsymbol{J}}$, i.e. the subspace spanned by vectors $d{\boldsymbol{\theta }}$ for which ${\boldsymbol{J}}d{\boldsymbol{\theta }} = \boldsymbol{0}$, and which leave the end-atom positions and orientations invariant, is generally *n*−5*m*-dimensional (Figure 1c). Vectors $d{\boldsymbol{\theta }}$ in this lower-dimensional space are *redundant* degrees-of-freedom that can be perturbed while maintaining distance constraints. Applying sufficiently small vectors from the null-space to a conformation will ensure that hydrogen-bond geometry is maintained. The right-singular vectors of the singular value decomposition ${\boldsymbol{J}}=\boldsymbol{U\Sigma V}^{\rm T}$ form a basis, ${\boldsymbol{N}}$, of the null-space of the Jacobian. A null-space perturbation projects a random trial-vector $\Delta {\boldsymbol{\theta }}$ onto the null-space, and adds it to the selected seed conformation: ${\boldsymbol{\theta }}_{{\rm new}} = {\boldsymbol{\theta }}_{{\rm seed}} + \boldsymbol{NN}^{\rm T} \Delta {\boldsymbol{\theta }}$. In contrast to techniques based on Laman constraint counting (19,22), our null-space method does not rely on the molecular conjecture (36) to identify exactly all rigid and flexible substructures in the molecule.

#### Rebuild perturbation

The sampling step size of a null-space perturbation is limited by the linearized forward kinematics. A rebuild perturbation allows for a larger step size, and accommodates sampling of preferred ribose conformations. A rebuild perturbation randomly selects a backbone segment of up to two nucleotides not constrained by base pairing or stacking interactions, and breaks the O3′–P bond at the 3′-end.

A new *τ*-angle is sampled for each ribose in the segment according to a bimodal probability distribution

\begin{equation*} P(\tau ) = 0.6 N_{\tau }(-154^{\circ },11.5^{\circ })+0.5N_{\tau }(44.7^{\circ },17.2^{\circ }) \end{equation*}

where *N_τ_* denotes the normal distribution, with peaks at the C3′-endo and C2′-endo conformations (Supplementary Figure S1). Glycosidic angles, *χ*, in the segment are resampled to a random value. After resampling, all backbone torsions of the segment, except those in riboses, starting at the P–O5′ bond at the 5′-end, are used to reclose the O3′–P bond. Reclosing of the segment is performed by iteratively applying the Moore–Penrose inverse of the Jacobian matrix, which, in linear approximation, minimizes the distance to reclose the O3′–P bond as a function of the backbone torsions.

### Sampling procedure

**Figure 2.**
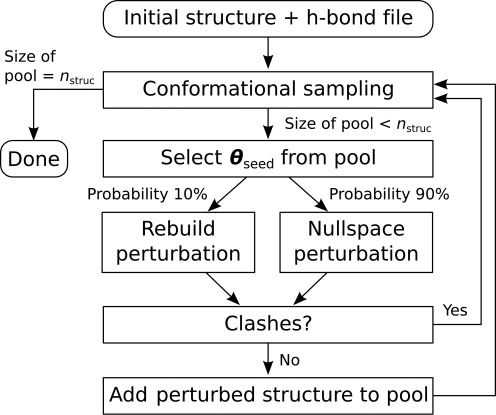
A flowchart of the KGSrna sampling algorithm. KGSrna takes as inputs an initial conformation and a file of hydrogen bonds A(N3)–U(H3) and G(H1)–C(N3) as distance constraints. Next, a pool of conformations is initialized with the input structure and then grown by repeatedly perturbing a randomly selected conformation from the pool with a rebuild or null-space perturbation at a 10/90 rate. If no clashes between atoms were introduced in the perturbed conformation, it is added to the pool. The procedure is repeated until a desired number *n*_struct_ of conformations is obtained.

An overview of the sampling procedure is shown in Figure 2. KGSrna takes as input an initial conformation $\boldsymbol{\theta }_{{\rm init}}$, an exploration radius *r*_init_ and a set of canonical WC pairs to identify hydrogen bonds A(N3)–U(H3) and G(H1)–C(N3) as distance constraints. WC pairs are obtained from the RNAView program (37). Next, it grows a pool of conformations by repeatedly perturbing either $\boldsymbol{\theta }_{{\rm init}}$ or a previously generated seed conformation, $\boldsymbol{\theta }_{{\rm seed}}$, in the pool that is within *r*_init_ C4′ root mean square deviation (RMSD) of $\boldsymbol{\theta }_{{\rm init}}$. The seed conformation is selected by first generating a completely random conformation $\boldsymbol{\theta }_{{\rm random}}$. Next, the conformation closest to $\boldsymbol{\theta }_{{\rm random}}$ from all previously generated conformations that are within a spherical shell of random radius from $\boldsymbol{\theta }_{{\rm init}}$ and width *r*_init_/100 is selected as $\boldsymbol{\theta }_{{\rm seed}}$, and then $\boldsymbol{\theta }_{{\rm random}}$ is discarded. This guarantees that samples in sparsely populated regions within the exploration sphere are more likely to be chosen as seeds and that the sample population will distribute widely. A rebuild perturbation of two free nucleotides or a null-space perturbation is then performed at a 10/90 rate. To characterize the apical loop of HIV-1 TAR, see below, the C2′-endo peak was up-shifted by 60° to oversample non-helical ribose conformations. A null-space perturbation can start from a seed generated by a rebuild perturbation or vice versa, allowing detailed exploration of remote parts of conformation space. The trial-vector is scaled down to ensure no torsional change exceeds 0.1 radians = 5.7°. If no clashes between atoms were introduced in generating a new sample, $\boldsymbol{\theta }_{{\rm new}}$, it is accepted in the conformation pool. An efficient grid-indexing method is used for clash detection by overlapping van der Waals radii (38). The van der Waals radii were scaled by a factor 0.5.

### Benchmark set

A benchmark set of 60 ncRNAs was compiled from the Biological Magnetic Resonance Bank (BMRB) (39) by selecting all single-chain RNA molecules that contain more than 15 nucleotides, were solved with NMR spectroscopy, and have measured chemical shift data available (Supplementary Table S1). Some molecules were removed to ensure that the edit-distance between the sequences of any pair was at least 5. To ensure uniformity in the benchmark set, the HPO_4_ group was removed at the 5′-end of the molecules 2LUB, 2LHP, 2AU4 and 2L94.

### Back calculating NMR properties from ensembles

Observed chemical shifts were obtained from the BMRB. Chemical shifts were back calculated using the software NUCHEMICS (40). A flowchart of the procedure used to obtain back calculated chemical shifts is shown in Supplementary Figure S1(a). RDC data were back calculated using the program PALES (41), with command line option -inD loopshort.tab -bestFit -pdb $file -H, where loopshort.tab contained measured RDC data for nucleotides 30–35 published by Dethoff *et al.* (42).

### Symmetric Kullback–Leibler divergence

The symmetrized KL divergence *D*_KL_(*P*‖*Q*) represents the difference between two discrete probability distributions *P* and *Q* (43). *D*_KL_(*P*‖*Q*) is often interpreted as the average number of bits of information lost when approximating one distribution by the other. It is defined as:

\begin{equation*} D_{{\rm KL}}(P \Vert Q) = \frac{D^{\prime }_{{\rm KL}}(P \Vert Q) +D^{\prime }_{{\rm KL}}(Q \Vert P)}{2} \end{equation*}

where $D^{\prime }_{{\rm KL}}(P \Vert Q) = \sum _i \ln (\frac{P(i)}{Q(i)})P(i)$.

To obtain the discrete probability distributions for a set of measured or predicted CS values, we created histograms with 30 bins in the range between the predicted mean ± four standard deviations. Small pseudocounts were added to empty bins to avoid a vanishing denominator in the divergence calculation, after which the distribution was renormalized.

### Fitting $^1\!{\boldsymbol{D}}_{{\rm CH}}$ residual dihedral coupling data

We calculated 20 000 Monte Carlo samples each starting from models 1 to 10 in the NMR bundle of wild type HIV-1 TAR with pdb id 1ANR. The sampling included the full, 29 nucleotide long models. Sampling was biased toward generically pairing the C30 and A35 bases and the U31 and G34 bases using a Metropolis criterion (Supplementary Figure S1(b)). A newly sampled structure was accepted into the pool of samples with a probability min (1, e^−Δ*d*^), where Δ*d* is the change from the seed to the new structure, i.e. $d = \min _{p\in P(30,35)}\left|2{{\rm A}}-|p| \right| + \min _{p\in P(31,34)}\left|2{{\rm A}}-|p| \right|$ and *P*(A, B) is the set of all vectors between any charged hydrogen in base A and any hydrogen acceptor in base B or vice versa. The seed selection was also modified. Instead of selecting a seed from the pool based on RMSD to the starting model it was based on Δ*d* from the starting model. For each of the 10 sets of 20 000 samples, we calculated RDCs with the software PALES. For each set, we fitted an optimal ensemble with a new algorithm we developed, called rdcFit, using the following quadratic program:

\begin{eqnarray*} \begin{array}{cl}\underset{w}{\text{min}} & \displaystyle \left\Vert ^1\!{\boldsymbol{D}}^{\rm o}_{{\rm CH}} - \sum _i w_i\;^1\!{\boldsymbol{D}}^{\rm c}_{{\rm CH},i}\right\Vert ^2 \\ \text{s.t.} & w_i \ge 0 \mbox{ for all } i \\ & 0 \le \displaystyle \sum _i w_i \le 1, \end{array} \end{eqnarray*}

where $^1\!{\boldsymbol{D}}^{\rm o}_{{\rm CH}}$ is a vector of observed RDCs and $^1\!{\boldsymbol{D}}^{\rm c}_{{\rm CH},i}$ a set of vectors of back-calculated RDCs for nucleotides 30–35. The vector ${\boldsymbol{w}}^{\rm T}$ is the fitted variable that simultaneously determines the optimal size of the ensemble and the relative weights of its members, under the constraint that the weights sum to unity. Unlike stochastic or heuristic optimization procedures, a constrained quadratic fit deterministically identifies the global optimum of the fitted parameters, i.e. both the size and weights of the ensemble.

To further optimize a transitional, ES-like state identified by the fitting procedure, 20 000 additional KGSrna Monte Carlo samples were calculated starting from the ES-like state. Our Metropolis criterion was restricted to hydrogen bonds C30(N4)–A35(N1), C30(N3)–A35(N6), U31(O2)–G34(N1) and U31(N3)–G34(O6) in this step to improve the ES-like state.

### Molecular dynamics simulations

All molecular dynamics simulations were performed with GROMACS 4.6.1 and the CHARMM 27 all-atom force field. The KGSrna structure was solvated in an octahedral unit cell with TIP3 water molecules and electrostatically neutralized by 28 Na ions (concentration 0.05 M and no ions within 6 Å of any RNA atom). The resulting system contained 13 054 water molecules and 40 120 atoms. For each of the 15 runs, the simulation system was minimized using a steepest descent algorithm, followed by a 150 ps MD equilibration applying a position restraint potential to the RNA heavy atoms. All simulations were run for 100 ns with constant NPT at a temperature of 300 K by coupling to a Nose–Hoover thermostat with a coupling constant of 0.6 ps and a Parrinello–Rahman barostat at a reference pressure of 1.0 bar. The van der Waals cutoff was set to 10 Å with a switching distance of 9 Å and the short-range electrostatics was set to 12 Å. Long-range electrostatic interactions were treated with the Particle-Mesh Ewald (PME) method. Non-bonded pair lists were updated every 10 steps with an integration step size of 2 fs in all simulations. All bonds were constrained using the LINCS algorithm.

### Availability

The KGSrna software is available at http://smb.slac.stanford.edu/∼vdbedem.

## RESULTS

### Accessing the native ensemble

**Figure 3.**
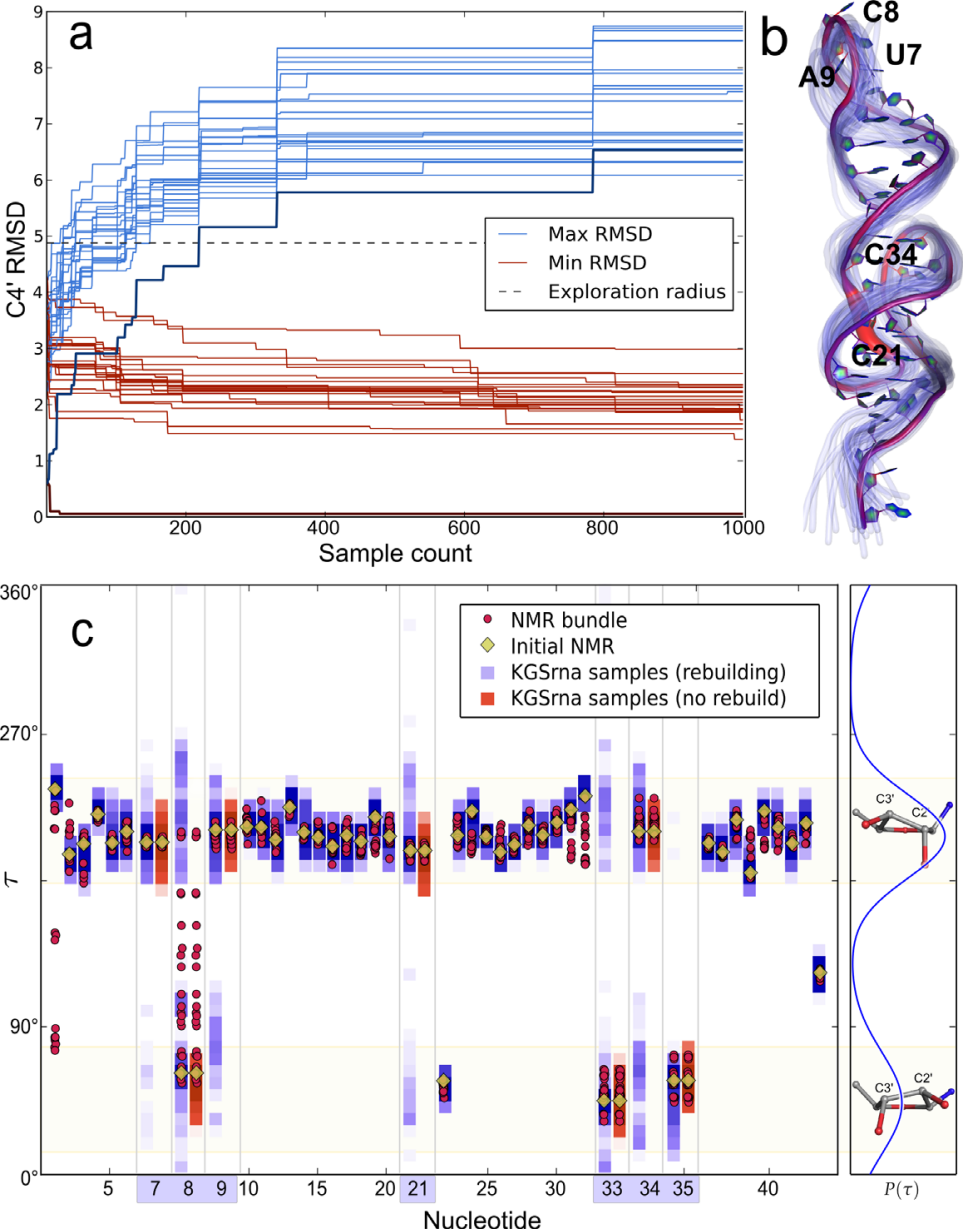
Sampling properties of 1000 KGSrna samples illustrated with the TYMV pseudoknot. Sampling was started from model one of the NMR bundle with pdb id 1A60, and the sampling-radius was set to 4.6 Å. (**a**) The evolution of the minimum (red curves) and maximum (blue curves) C4′ RMSD to each structure in the NMR bundle. Bold curves correspond to the starting structure. (**b**) The backbone of the initial structure indicating varying degrees of ridigity for torsional degrees-of-freedom. A thicker and more red-shifted backbone indicates higher variances for those degrees-of-freedom. The backbones of 25 randomly chosen KGSrna samples are shown in translucent blue to reflect flexibility. (**c**) Distributions of the *τ*-angles in the NMR-bundle and the KGSrna samples. Ribose conformations of the 1000 samples are displayed vertically as normalized color-coded histograms with a bin-width of 1.8°. Rebuild perturbations recover the full range of *τ*-angles in the NMR bundle for free nucleotides (highlighted on the *x*-axis), as shown by residue 8. The distribution from which *τ*-angles are sampled is shown on the right. The large peak corresponds to C3′-endo conformations and the smaller one to C2′-endo conformations.

Efficient exploration of the native ensemble requires broad and uniform sampling. Sampled conformations need to diffuse away quickly from an initial structure, while simultaneously at least one member of the native ensemble should be found close to any sampled conformation. We first validated these characteristics for KGSrna on a benchmark set of 60 RNA molecules with an average length of 30 nucleotides (nt) determined by NMR spectroscopy from the BMRB (Supplementary Table S1). We view the NMR bundle as structural representatives of a native ensemble, i.e. a ‘synthetic’ ensemble. For each RNA molecule, we created a set of 1000 samples starting from the first model of the NMR bundle. The exploration radius was fixed at the largest pairwise RMSD in each NMR bundle. Creation of 1000 samples took on average 372 s. Figure 3a shows the evolution of the C4′ RMSD between 1000 KGSrna samples and the NMR bundle of the 44 nt pseudoknotted acceptor arm of the transfer RNA-like structure of turnip yellow mosaic virus (TYMV). The procedure quickly expands its sampling neighborhood from the starting model to exceed its preset exploration radius of 4.9 Å (Figure 3a bold blue line). Within ∼300 sampling steps, the distance to the starting model reaches a limiting distance of ∼1.5 Å beyond the exploration radius, a trend that was consistent across our benchmark set (Supplementary Table S1). The maximum RMSD to each member of the NMR bundle of the sample set, represented by the blue lines, ranges from 6.1 to 8.7 Å. These trends indicate that samples diffuse quickly and uniformly through the synthetic ensemble, away from the starting model and consistently equidistant to all members of the NMR bundle.

Each member of the NMR bundle is closely approximated by a KGSrna sampled conformation as shown by the minimum RMSD (red lines). For the TYMV pseudoknot, the minimum RMSD also converges rapidly to a limiting value of ∼2 Å. This trend is also consistent across our benchmark set, with an average minimum RMSD of 1.2 Å (Supplementary Table S1). These metrics compare favorably to RNA 3D structure prediction algorithms (44), which suggests that our procedure can be used as an efficient conformational search procedure to further refine *ab initio* structures.

Regions of the molecule that are highly constrained by hydrogen bonds are difficult to deform, which is intrinsic in our kinematic representation by sharply reduced trial deviations after projecting into the null-space. In Figure 3b we color-coded the backbone of the TYMV pseudoknot to reflect the degree of rigidity for each backbone degree-of-freedom. The acceptor arm of TYMV contains two loops. Loop I (C21–U24), which spans the major groove, is clearly identifiable with highly flexibly degrees-of-freedom and loop II (U33–A35), which spans the minor groove, also stands out. The T loop, at the 3′-end, is somewhat less flexible. The variance in atom positions is illustrated by 25 randomly chosen KGSrna samples, shown in translucent blue. While conformational heterogeneity appears to be concentrated around the 3′- and 5′-end of the molecule, it originates primarily from the three least constrained backbone regions, loops I and II, and the T loop.

We then examined if rebuilding segments while sampling preferred ribose conformations would accurately represent ribose conformations of the NMR bundles. While C3′-endo to C2′-endo conformational transitions are rare in double strand regions, they are expected to occur more frequently in loop regions (45). In the benchmark set only 4.3% of all nucleotides occur with both C3′-endo and C2′-endo ribose conformations in the NMR bundle, but among unconstrained loop residues there are 35%. Ribose conformations can have important long-range structural effects, changing helical conformations and playing critical roles in binding events (46).

Unsurprisingly, rebuilding of unconstrained segments results in broader sampling of the ribose conformation. Starting from the first model of the NMR bundles, only nine out of 196 ribose conformations (4.8%) with both C3′-endo and C2′-endo are fully recovered using null-space perturbations. In contrast, rebuild perturbations recover all but four ribose conformations (98%). These four are all in less common conformations such as O4′-endo or C1′-endo. Figure 3c shows the range of *τ*-angles sampled by each residue from the TYMV pseudoknot obtained from null-space perturbations only (magenta squares) and with rebuild perturbations enabled (blue squares). Without rebuild perturbations, sampled *τ*-angles remain close to their initial values obtained from the first model of the NMR conformational ensemble (yellow diamonds). For nucleotide C8 for example, null-space perturbations are unable to recover the full range of *τ*-angles in the NMR bundle (red circles), but rebuild perturbations do.

### KGSrna recovers proton chemical shifts

Chemical shifts are time-averaged measurements on conformational ensembles at sub-millisecond timescales (23). Non-exchangeable ^1^H chemical shifts (CS) predicted directly from RNA 3D structural models are generally in excellent agreement with those reported from experiments in the BMRB. Experimental ^1^H CS are widely available, are sensitive to conformational changes and have aided in structurally characterizing conformational substates (47). Researchers have combined measured ^1^H CS for proteins with structure prediction algorithms that use a database of structural fragments to determine atomically detailed *de novo* conformations (48). Das *et al.* recently established that proton chemical shifts can aid structure prediction algorithms in distinguishing decoys from a native state in RNAs (26).

Here, we regard measured ^1^H CS on a benchmark set of 3D RNA structures as time- and ensemble-averaged distributions over the conformational landscape. We examined the ability of KGSrna to sample native dynamical ensembles that recover sugar (H1′) and nucleobase (H2, H5, H6 and H8) CS distributions for unconstrained (non-helical) and WC paired (helical) regions. We used the well-calibrated program NUCHEMICS (40) to predict ^1^H CS from our 3D RNA structures.

**Figure 4.**
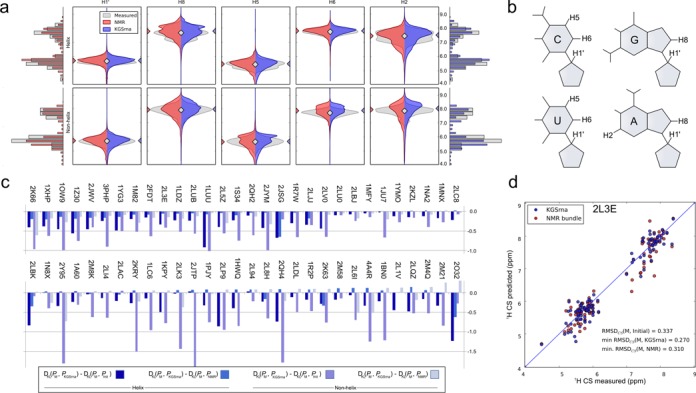
Agreement between measured ^1^H chemical shifts and those back calculated from KGSrna and NMR 3D structures. (**a**) Depicted chemical shift values are aggregated by proton type in helical (top) and non-helical regions (bottom). The discrete distributions were smoothed with a Gaussian kernel density estimator (bandwidth *n*^−0.2^ where *n* is number of data points) for easier visualization. Measured values were taken from the BMRB and KGSrna samples and NMR bundle values were back calculated using NUCHEMICS. Marginal distributions are shown as histograms with bin-widths of 0.275 ppm. (**b**) The symmetric Kullback–Leibler divergence indicates the degree of similarity of two distributions and is calculated for the marginal distributions of measured-to-KGSrna, measured-to-initial and measured-to-NMR. The differences between measured-to-KGSrna, measured-to-initial and measured-to-NMR are shown in the bar plot. A negative value indicates better agreement of the ensemble of KGSrna 3D structures with measured values than its comparison 3D structures. (**c**) Predicted ^1^H chemical shifts calculated from the 3D structures from the KGSrna ensemble and the NMR bundle compared to the measured values of the 32nt P2a-J2a/b-P2b (helix-bulge-helix) of human telomerase RNA (pdb id 2L3E). The data points are expected to lie along a 45° line if measured ^1^H CS are accurately predicted.

KGSrna enables broad sampling to identify sparsely populated substates, while maintaining conformational distributions similar to those measured. Figure 4a shows the distribution of measured and predicted ^1^H CS for helical (top row) and non-helical (bottom row) regions for each proton type over the whole benchmark set. Figure 4b shows the location of the probes. For helix backbone and base protons, the medians of the distributions are virtually identical. This suggests that, on average, our kinematic representation of RNA results in an unbiased exploration of the conformational landscape encoded in the measured proton chemical shifts.

For helical and non-helical regions, aggregate and individual (Supplementary Figure S1) sampling distributions of ^1^H CS obtained with KGSrna are visually similar to the distributions obtained from experimental measurements. To further compare similarities between the measured chemical shift distributions *P*^M^ and the predicted distribution *P*^KGSrna^, we calculated the symmetrized Kullback–Leibler (KL) divergences *D*_KL_(*P*^M^‖*P*^KGSrna^) of *P*^KGSrna^ from *P*^M^ and compared those to the KL divergences *D*_KL_(*P*^M^‖*P*^init^) and *D*_KL_(*P*^M^‖*P*^NMR^) for our benchmark set (Figure 4c; Supplementary Table S1; Materials and Methods). The distributions *P*^init^ and *P*^NMR^ are the predicted distributions calculated from the first model of the NMR bundle only and the full NMR bundle. Both *P*^KGSrna^ and *P*^NMR^ deviate from *P*^M^, in part owing to weighted motional averaging of the measured shifts. Similarities between the KGSrna predicted distributions and the measured distributions exceeded those of the predicted distributions from the first model. The distribution of chemical shifts contributed by helical regions is expected to diverge less widely than that contributed from non-helical regions, owing to restrained conformational diversity. However, KGSrna predicted chemical shifts for both regions to a similar, accurate degree compared to a single starting model or the full NMR bundle. In 58 out of 60 of cases, KGSrna improved agreement with the distribution of measured ^1^H CS in non-helical regions (58 out of 60 for helical regions too) compared to the distribution calculated from the first model. The average KL divergence reduction was 37% (42% for helical regions (Supplementary Table S1, Figure 4c). This suggests that KGSrna is able to diverge from the starting model, and explores beyond a local neighborhood of conformational space. In addition, in 70% of cases KGSrna improved agreement with the distribution of measured ^1^H CS in non-helical regions (58% for helical regions) compared to the distribution calculated from the full NMR bundle. Predictions for non-helical regions were improved by our rebuilding procedure, conceivably resolving structural disorder inadequately represented by the NMR bundle (40). The similarities between predicted and measured distributions suggest that a simple kinematic model with constraints samples the conformational landscape according to the same distribution as RNA in solution.

We then asked how accurately just a single KGSrna sample could recover measured chemical shifts. The error between measured and predicted CS is attributable to measurement errors and systematic errors in prediction. Additionally, measured chemical shifts are a weighted motional average. We therefore regarded the NMR 3D conformer that best agrees with measured chemical shifts as a benchmark of predictive value.

We calculated the RMSD (RMSD_CS_) between the measured and predicted chemical shifts for all proton types for each 3D model in the NMR bundles and in the KGSrna sample sets (Supplementary Table S1). The minimum RMSD_CS_(M, KGSrna) ranges from 0.17 to 0.54 ppm (mean 0.30 ppm) and the minimum RMSD_CS_(M, NMR) from 0.16 to 0.53 ppm (mean 0.30 ppm). A recent study observed a mean minimum weighted RMSD_CS_ of 0.23 ppm (ranging from 0.16 to 0.35 ppm) for an ensemble of 8000 conformers obtained from molecular dynamics simulations for four RNAs, but the proton chemical shifts were weighted to favor those that better agreed with measured values (23). In 80% of cases in our benchmark set, the RMSD_CS_ of the best KGSrna conformer is lower than that of the best conformer identified from the NMR bundle (Figure 4d). The average improvement over the starting model is 18% (*p*-value < 0.01), and in some cases exceeds 40%. As proton chemical shifts can discriminate a native state, this result suggests that a simple kinematic representation yields a powerful conformational search algorithm.

### KGSrna reveals a hairpin loop excited state from $^1\!{\boldsymbol{D}}_{{\rm CH}}$ HIV-1 TAR data

The 5′-end of the human immunodeficiency virus type-1 (HIV-1) transcript contains a 59-nucleotide trans-activation response element (TAR) stem–loop (49). In the ground state, HIV-1 TAR binds human cyclin T1 and viral trans-activator protein Tat that activate and enhance transcription of the HIV-1 genome (42,50–52). The HIV-1 TAR apical hairpin loop plays a key role in binding Tat. Available structures for the HIV-1 TAR apical loop exhibit significant conformational differences, which indicate that the loop is highly flexible. However, a full atomic characterization of the structure and dynamics of the HIV-1 TAR hairpin loop remains elusive. Al-Hashimi *et al.* recently proposed a two-state model (ground and excited state, GS and ES) of the apical HIV-1 TAR hairpin loop from NMR *R*_1ρ_ relaxation dispersion measurements and mutagenesis (53). Their study suggested formation of a U_31_G_32_G_33_G_34_ tetraloop in the ES, with a non-canonical closing base-pair C30–A35.

RDCs report the amplitude of motions that reorient C-H and N-H bond vectors on the sub-millisecond time-scale. Experimentally observed RDCs are a weighted average of all conformational substates. ‘Sample-and-select’ strategies, which rely on generating a large number of samples from which a subset is selected that best explains experimental data, have previously led to insights into conformational dynamics and functional mechanisms in X-ray crystallography and NMR data (54–56).

KGSrna was designed to explore correlated conformational variability resulting from nested, closed rings. The degrees-of-freedom of the HIV-1 TAR hairpin apical loop participate in the nested, closed rings formed by the canonical base-pairs in the stem. To test if KGSrna can structurally characterize conformational substates of the loop guided by RDC data, we calculated 20 000 samples each starting from the full, 29 nucleotide models 1 to 10 in the NMR bundle with pdb id 1ANR of free HIV-1 TAR. To enable structural characterization of the dynamics leading to the ES, we biased our sampling toward broad, non-specific conformational pairing of C30–A35 and U31–G34. A Metropolis criterion skewed the sample set to include favorable interactions of any charged hydrogen in base A with any hydrogen acceptor in base B (Materials and Methods). For each of the 200 000 samples, we back-calculated RDCs with the program PALES (41). PALES accurately calculates the overall alignment of the RNA molecule (18). From each batch of 20 000, we then determined a weighted ensemble that optimally explained the experimentally observed RDCs using a new constrained quadratic fit algorithm (rdcFit) that we adapted from an application we previously developed for X-ray crystallography applications (qFit) (54,55).

**Figure 5.**
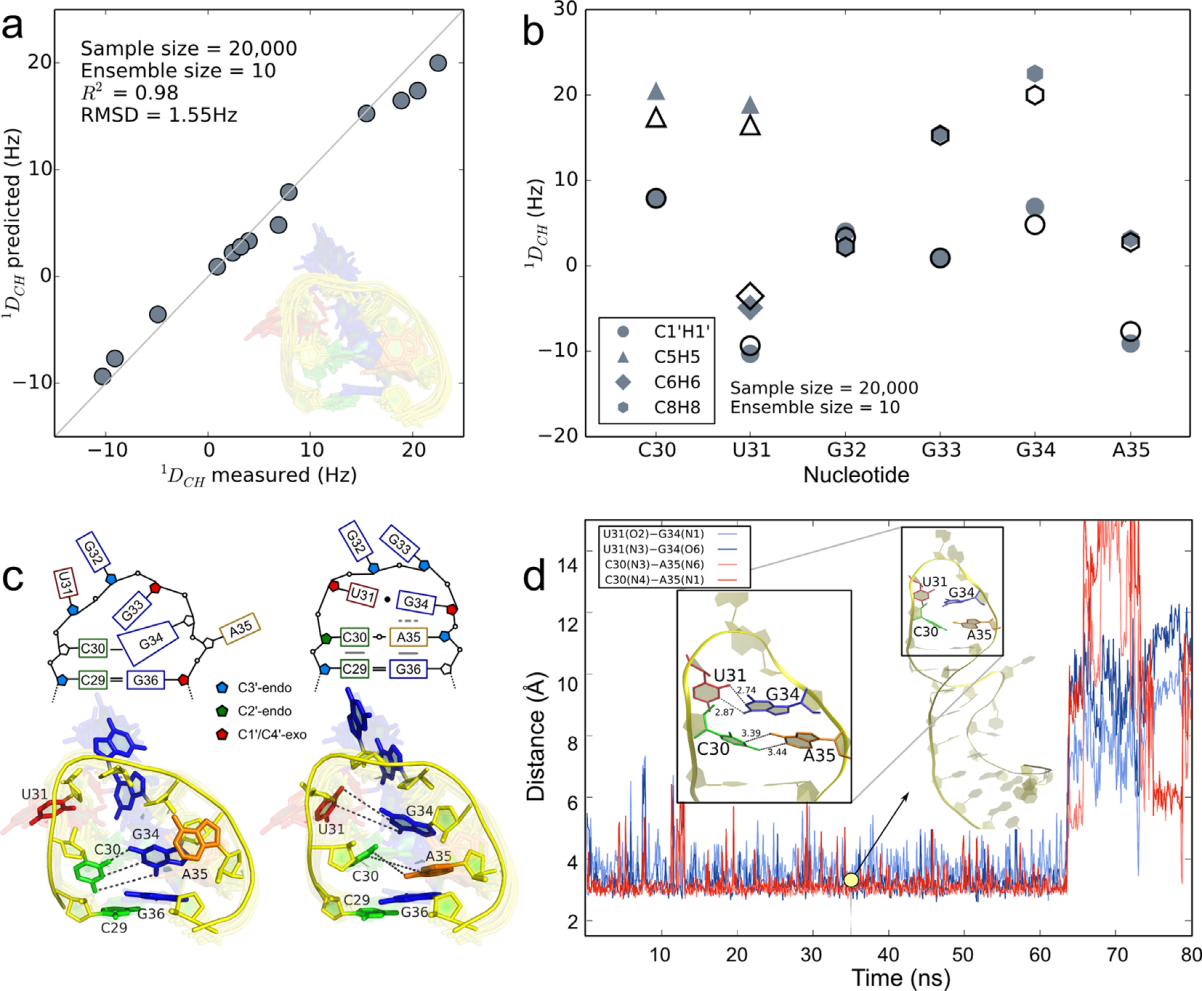
Structural characterization of conformational substates of the apical Tat binding loop of HIV-1 TAR. (**a**) Paired measured RDCs for apical loop nucleotides and those predicted from a 10-member weighted ensemble (inset) obtained from fitting 20 000 KGSrna samples to measured RDCs with a quadratic program. The data points are expected to lie along a 45° line if measured RDCs are accurately predicted. The coefficient of determination for the predicted RDCs equals 0.98. (**b**) Observed (solid symbols) and predicted (open symbols) RDCs for apical loop nucleotides. Smaller magnitudes for RDCs generally indicate more angular mobility in the bond vectors. (**c**) Schematic of the GS (top panel, left) and the ES (top panel, right) corresponding to the 3D structures closest to the GS and the ES in the 10-member ensemble. The bottom left panel shows the GS highlighted in the ensemble, with the other members translucent in the background. The bottom right panel shows the ES identified from biasing the sampling toward pairing C30–A35 and U31–G34. (**d**) Time evolution of the hydrogen bond distances between reverse wobble pair C30–A35 (blue colors) and GU wobble pair U31–G34 (red colors) in the ES of HIV-1 TAR for 80 ns of the molecular dynamics trajectory. The distances shown are between heavy donor and acceptor atoms, sampled every 100 ps. Along the trajectory, the apical loop maintains a helical structure (inset at 35 ns) until, at 65 ns, pairing of U31–G34 and subsequently C30–A35 is disrupted.

This procedure identified a 10-member, weighted ensemble from the sample set starting from model seven in the NMR bundle that agrees extremely well with experimentally observed RDC values (Figure 5a). The coefficient of determination between observed $^1\!{\boldsymbol{D}}_{{\rm CH}}$ values and those predicted from the weighted ensemble equals 0.98. The predicted values of the ensemble accurately reflect the mobility of riboses and nucleobases, with $^1\!{\boldsymbol{D}}_{{\rm CH}}$ small in magnitude indicating elevated mobility (Figure 5b). The RMSD between observed and predicted $^1\!{\boldsymbol{D}}_{{\rm CH}}$ values is 1.55 Hz, below the experimental error of 2–4 Hz (16,18).

Our ensemble characterizes disparities in mobility between nucleotides in exquisite atomic detail, consistent with the RDC data (Figure 5a inset). In our ensemble nucleobases U31, G32 and A35 are most mobile, with motions indicating looping in and out. Small magnitudes of U31 $^1\!{\boldsymbol{D}}_{{\rm C}6{\rm H}6}$, G32 $^1\!{\boldsymbol{D}}_{{\rm C}8{\rm H}8}$, and A35 $^1\!{\boldsymbol{D}}_{{\rm C}8{\rm H}8}$ experimental values support this interpretation (Figure 5b). G34 exhibits a more limited range of motion in the ensemble, consistent with larger values of G34 $^1\!{\boldsymbol{D}}_{{\rm C}8{\rm H}8}$. G32 is more mobile than G33, and is looped out for all members of the ensemble. Stacking interactions reported in MD simulations between U31 and G32 (42) or G32 and G33 (49) are not represented in our ensemble. A previously reported and experimentally confirmed base triple (A22–U40)U31 (47) was not observed in our ensemble.

In the conformation of our ensemble most closely exhibiting features attributed to the GS, we confirmed the formation of a stabilizing cross-loop WC bp C30·G34 (49) (Figure 5c, left). Nucleobases A35 and U31 are looped out in this conformation, while G33 is in front of the loop, possibly interacting with C30 and G34. A second conformation of our ensemble exhibits features closely associated with a transition to the ES, suggesting that C30 and A35 are poised to adopt a reverse wobble pair, with hydrogen bonds C30(N4)–A35(N1) and C30(N3)–A35(N6). G34 is positioned to adopt a GU wobble pair with U31 through hydrogen bonds U31(O2)–G34(N1) and U31(N3)–G34(O6) (57) (Figure 5c, right).

The G34 glycosidic angle in our ensemble is (high) anti, ranging from −110.2° to −83.7°. In the GS of our ensemble, G34 adopts an anti base (−93.2°), while it is high anti (−83.7°) in the transition to ES. In the formation of UUCG tetraloops, it is common for the guanine to loop out to accommodate a transition from anti to syn (58). Experimental $^1\!{\boldsymbol{D}}_{{\rm C}8{\rm H}8}$ data do not appear to support a similar large amplitude motion of the G34 base when adjusting from anti to syn. Instead, our ensemble suggests that G34 gently readjusts to accommodate A35 looping in.

To confirm this intermediate state toward the ES, we generated an additional 20 000 samples starting from this conformation, instructing KGSrna to further optimize the CA reverse wobble and the GU wobble pairs with the Metropolis criterion (Materials and Methods). In the model with most ideal hydrogen-bond geometry between these bases, we observe ribose conformations suggesting that C30 and A35 are adopting a C3′-endo conformation, continuing the A-form helical stem from bp C29·G36. To examine if the ES is kinetically accessible from this intermediate state, we started 15 independent, 100 ns molecular dynamics (MD) simulations (see ‘Materials and Methods’ section).

Consistent with the transient character of the ES, the CA reverse wobble pair and GU wobble pair were maintained for 20–65 ns in duration for 4 out of 15 or 27% of the MD simulations. In the remaining simulations, pairings did not occur or were short-lived. Figure 4d shows the evolution of the distances of the hydrogen bonds between the CA pair and the GU pair for the MD simulation that maintained pairing for nearly 65 ns. The GU pair interacts more weakly than the CA pair, with U31 staggered toward the apex of the loop (Supplementary Figure S2). G34 forms additional hydrogen bonds G34(N2)–U31(O4′) and G34(N1)–C30(O2′) that stabilize the ES (Supplementary Figure S2). In the independent MD simulations, looping out of U31 generally disrupts its pairing with G34. Subsequently, the reverse wobble CA pair is disrupted. The helical conformation is extended in the ES; the riboses of G34 and A35 largely adopt C3′-endo conformations in the simulation. The riboses of C30 and U31 adopt a C2′-endo conformation for the duration of U31–G34 pairing, after which C30 adopts a C3′-endo conformation (Supplementary Figure S3). G32 and G33 intermittently stack during the simulation (Figure 5d inset). To our knowledge, this is the first time sustained and simultaneous pairing of C30–A35 and U31–G34 observed in MD simulations of HIV-1 TAR.

## DISCUSSION

Molecular dynamics simulations can often provide new and highly detailed insight into specific, atomic interactions and functional mechanisms of biomolecules. By contrast, recent advances suggest that random sampling algorithms, coupled with knowledge-based potentials (33) and/or sparse experimental data (26), are better suited to provide broad exploration of the conformational landscape. Our analysis demonstrates that conformational ensembles of non-coding RNAs in solution can be accessed from efficiently sampling coordinated changes in rotational degrees-of-freedom that preserve the hydrogen bonding network. Compared to exploring the conformational landscape with molecular dynamics simulations, our highly simplified structural representation obtains similar agreement between measured and predicted chemical shifts from fewer samples and unweighted RMSD. Coordinated changes enforced by the kinematic representation deform the molecule along preferred directions on the conformational landscape, overlapping with those avoiding hydrogen bond dissociation. These intrinsic constraints on deformation enable our procedure to efficiently probe the conformational diversity resulting from equilibrium fluctuations of the ensemble, suggesting that a kinematic representation is capable to encode the dominant forces within a folded polynucleotide corresponding to sub-millisecond, RDC time scales.

Our procedure directly encodes rigidity of RNA molecules. By analyzing how flexibility propagates through amino acids in room temperature X-ray diffraction data we previously established that 3D networks related to functional mechanisms partition protein molecules (55). These networks can provide important mechanistic insights into binding events and the role of allostery in activation. While a purely kinematic model does not suffice to determine strain, i.e. the deformation of a biomolecule due to stress, kinematics can elucidate long-range effects of locking or unlocking degrees-of-freedom through mutations and altered non-covalent bonds.

Combined with experimental data, KGSrna enables structural biologists to quickly formulate and test hypothesis about conformational dynamics, and offers tremendous potential for uncovering functional mechanisms. Our integrative analysis of HIV-1 TAR, linking structural experimental data and relaxation dispersion data with advanced computational algorithms, enabled us to identify an intermediate state that relaxes to the ES. However, the detailed structure of ground and evanescent excited states and their precise transitional mechanisms remain unresolved. While other researchers have posited a U31–G34 reverse wobble base-pair based on analogy with UUCG tetraloops and downfield shifted chemical shifts, we find that the anti-G34 base suggests a staggered U31–G34 wobble pair in the excited state. A mechanism analogous to the formation of UNCG tetraloops proposed for the HIV-1 TAR hairpin ES is not supported by our structural analysis of RDC data (53).

The set of feasible conformations for the apical loop of HIV-1 TAR is huge. While a convex quadratic fit of predicted to experimentally observed RDCs provably determines the global optimum, the quality of that optimum is limited by conformational sampling. The fitted ensemble approximates true substates, which, averaged, constitute the NMR measurements. While it is tempting to associate fractional contributions with population lifetimes, we should expect those to compensate for any conformational inaccuracies. A more direct correspondence between fractional contributions and population lifetimes will require bounding conformational space and/or additional data (54,55).

Our results suggest that diffusive motions of RNA are restricted to a lower-dimensional subspace of conformation space. As secondary structure prediction algorithms for RNA have matured greatly, this insight can have important implications for the efficiency and accuracy of search methods in 3D structure prediction and biomolecular docking applications. These methods rely on coarse-grained representations, which are often derived heuristically (31). By contrast, our null-space procedure naturally reduces the dimensionality of the system. It automatically partitions the molecule into ’free’ and ’cycle’ degrees-of-freedom, of which only the latter require coordinated changes.

NMR relaxation dispersion experiments can provide highly detailed insight into transient, sparsely populated substates, but high energetic barriers frequently prevent access through molecular dynamics simulations. Our method provides a widely applicable, new avenue to uncover RNA conformational diversity from a variety of data sources. Combined with mutagenesis, our new approach can be used to relate motion to function with implications for RNA engineering and drug design.

## SUPPLEMENTARY DATA

Supplementary Data are available at NAR Online.

SUPPLEMENTARY DATA
