# Supplementary material for: Characterizing RNA ensembles from NMR data with kinematic models
Source: Nucleic Acids Res. 2014 Aug 11;42(15):9562–72. doi: 10.1093/nar/gku707 (PMC4150802; doi:10.1093/nar/gku707)
Supplement: SUPPLEMENTARY DATA [file supp_42_15_9562__index.html]

Characterizing RNA ensembles from NMR data with kinematic models — SUPPLEMENTARY DATA 

# Characterizing RNA ensembles from NMR data with kinematic models

## SUPPLEMENTARY DATA

**Files in this Data Supplement:**

- Supplemental Figures and Tables
- Supplemental Data 1
- Supplemental Data 2
- Supplemental Data 3
