# Supplementary material for: Characterizing RNA ensembles from NMR data with kinematic models
Source: Nucleic Acids Res. 2014 Aug 11;42(15):9562–72. doi: 10.1093/nar/gku707 (PMC4150802; doi:10.1093/nar/gku707)
Supplement: SUPPLEMENTARY DATA [file supp_gku707_nar-01345-z-2014-File012.pdf]

Supplementary table and figures to: Characterizing RNA ensembles from NMR data with kinematic models

Rasmus Fonseca, Dimitar V. Pachov, Julie Bernauer, and Henry van den Bedem

Supplementary Table 1. Characteristics of the benchmark set. The 60 RNA molecules represent all non-redundant single-chain structures from the BMRB with more than 15 residues and available chemical shift data. Column 4: A pseudo-knot topology is indicated with a P and bridge-topologies with B. Column 5: The exploration radius is the largest RMSD between two NMR bundle structures and is used as a cutoff for seed-selection in the sampling procedure. The min $RMSD_{NMR}$ is the smallest C4’ RMSD between any member of the KGSrna samples and an NMR bundle structure. The average over all NMR bundle structures is reported in column 6. Columns 7 to 9 show the symmetric Kullback-Leibler divergence between measured chemical shift distributions and predicted distributions for KGSrna, initial structure and entire NMR bundle helical protons respectively. Columns 10 to 12 are the same only for non-helical protons. Columns 13 to 15 indicate the minimum RMSD between measured proton chemical shifts and KGSrna generated structures, the NMR bundle, and the initial structure, respectively. Bold typesetting indicates the minimum value in each row.

						Helix			Non-helix					
PDB-id	Description	Length	Topology	Exploration radius	Avg. min $RMSD_{NMR}$	$D_{KL}(M,KGSrna)$	$D_{KL}(M,init)$	$D_{KL}(M,NMR)$	$D_{KL}(M,KGSrna)$	$D_{KL}(M,init)$	$D_{KL}(M,NMR)$	min $RMSD_{CS}(M,KGSrna)$	min $RMSD_{CS}(M,NMR)$	$RMSD_{CS}(M,init)$
2K66	d3' stem of group II intron Sc.ai5γ	23		1.1	0.3	**0.51**	0.92	0.71	**0.93**	1.90	1.54	**0.36**	0.46	0.47
1XHP	Extendd U6 ISL	33		0.8	0.3	**0.35**	0.73	0.55	**0.61**	1.23	0.79	0.25	**0.25**	0.27
1OW9	Active conf. of VS ribozyme	24		2.3	0.5	**0.58**	1.04	0.72	**0.66**	1.65	0.95	**0.28**	0.46	0.54
1Z30	Stemloop D from BEV	19		1.3	0.4	**0.49**	1.05	0.68	**0.39**	1.15	0.54	**0.31**	0.36	0.42
2JWV	High affinity anti-NFkB RNA aptamer	30		3.4	0.9	**0.49**	0.95	0.65	**0.68**	1.09	0.81	**0.25**	0.26	0.27
3PHP	3' hairpin of TYMV pseudoknot	24		4.9	1.4	**0.48**	0.72	0.64	**1.07**	1.97	1.18	**0.23**	0.23	0.27
1YG3	ScYLV P1-P2 frameshifting pseudoknot	29	P	2.7	1.2	**0.26**	0.74	0.40	**0.49**	0.99	0.58	**0.35**	0.40	0.40
1M82	cRNA promoter of influenza A	26		3.0	1.2	**0.33**	0.53	0.42	**0.82**	1.34	1.12	0.24	**0.22**	0.26
2FDT	Hairpin of eel LINE UnaL2	37		1.8	0.8	**0.24**	0.40	0.32	**0.39**	1.02	0.55	**0.20**	0.21	0.21
2L3E	P2a-J2a/b-P2b of human telomerase	36		3.5	1.1	**0.26**	0.59	0.31	**0.43**	0.85	0.58	**0.27**	0.31	0.34
1LDZ	Lead-dependent ribozyme	31		7.8	1.8	**0.70**	0.94	0.76	**0.62**	1.15	0.81	**0.30**	0.34	0.35
2LUB	Helix H1 of human HAR1	38		2.3	1.0	**0.28**	0.51	0.33	**0.54**	1.11	0.66	**0.29**	0.34	0.34
1LUU	ASL of yeast tRNA-PHE	18		1.4	0.4	**0.78**	1.70	0.85	**1.59**	2.60	1.64	**0.31**	0.32	0.42
2L5Z	A730 loop of Neurospora VS ribozyme	27		1.3	0.5	**0.45**	0.85	0.49	**0.60**	1.16	0.90	**0.28**	0.33	0.34
1S34	Splice site of Rous sarcoma virus	24		2.6	1.2	**0.48**	0.71	0.58	**1.03**	1.78	1.07	0.23	**0.19**	0.29
2QH2	CR7 term hairpin from human telom.	25		1.9	0.8	**0.36**	0.47	0.39	**1.66**	2.26	1.79	0.25	**0.25**	0.26
2JYM	Stemloop A of HBV HPRE	23		1.8	0.7	**0.66**	1.08	0.69	**1.77**	2.76	2.10	**0.31**	0.32	0.37
2JSG	Anticodon of E.coli TRNA-VAL3	18		1.1	0.4	**0.62**	1.30	1.28	**1.02**	1.22	1.04	**0.26**	0.29	0.35
1R7W	D4 stem-loop B of enterovirus IRES	35		8.8	2.9	**0.21**	0.51	0.25	**0.96**	1.62	0.98	**0.19**	0.22	0.24
2LJJ	Subdom. IV-B from CVB-3 IRES	28		2.7	1.1	**0.71**	1.06	0.89	**0.95**	1.30	0.96	**0.39**	0.46	0.54
2LV0	Helix-35 stemloop of e.coli 23S rRNA	25		2.9	0.9	**0.69**	1.13	0.70	**0.92**	1.57	1.01	**0.24**	0.28	0.29
2LU0	κ-ζ reg. of group II intron Sc.ai5γ	50		10.0	3.2	**0.20**	0.30	0.21	**0.26**	0.35	0.31	**0.33**	0.37	0.48
2LBJ	ASL of b.subtilis tRNA-GLY	18		1.0	0.4	**0.59**	0.66	0.59	**1.09**	1.33	1.27	**0.31**	0.34	0.44
1MFY	C4 promoter of influenza A	32		4.1	1.5	**0.78**	0.83	0.79	**0.40**	0.55	0.45	0.30	**0.30**	0.36
1JU7	SLBP binding site	17		2.2	0.7	0.67	0.70	**0.67**	**0.87**	1.54	1.07	**0.22**	0.27	0.29
1YMO	P2b-P3 p.knot from human telomerase	48	P	2.4	1.0	0.28	0.40	**0.27**	**0.24**	0.52	0.31	**0.41**	0.42	0.47
2KZL	GA motif of B. subtilis tyrS T box leader	56		2.4	1.1	0.55	0.70	**0.54**	**0.44**	0.60	0.44	**0.44**	0.46	0.47
1NA2	P2B hairpin from human telomerase	31		2.4	0.9	0.33	0.45	**0.31**	**0.74**	1.14	0.82	**0.22**	0.30	0.30
1MNX	Loop region of 5S rRNA	43		2.3	1.1	0.27	0.44	**0.27**	0.29	0.56	**0.27**	**0.26**	0.29	0.30
2LC8	MLV readthrough pseudoknot	57	P	5.1	2.3	0.51	0.73	**0.49**	**0.76**	0.84	0.80	**0.36**	0.36	0.37
2LBK	ASL of s.epidermis tRNA-GLY	18		1.5	0.5	**1.02**	1.86	1.36	0.71	0.80	**0.69**	**0.21**	0.21	0.22
1N8X	HIV1 stem loop SL1	37		3.4	1.2	0.32	0.33	**0.29**	**0.84**	1.24	1.10	**0.26**	0.29	0.30
2Y95	AUCG tetraloop human Xist A-repeat	15		1.3	0.4	0.77	1.12	**0.74**	**1.34**	3.14	2.07	**0.16**	0.17	0.20
1A60	T and acceptor arm of TYMV	45	P/B	4.9	2.0	0.47	0.70	**0.43**	**0.50**	0.81	0.59	**0.37**	0.39	0.40
2M8K	Pyr motif triple helix of k.lactis telomerase	49	P	1.9	0.9	**0.28**	0.33	0.32	0.85	1.48	**0.82**	**0.31**	0.34	0.34
2LI4	Antiterminator from Mg2+ riboswitch	33		7.7	2.1	**0.38**	0.52	0.39	0.72	1.36	**0.67**	0.28	**0.26**	0.33
2LAC	ASL of b.subtilis tRNA-TYR	18		0.7	0.3	**1.10**	1.32	1.16	1.06	**1.01**	1.09	**0.27**	0.28	0.29
2KRY	Mitochondrial tRNA-MET ASL from human	17		2.6	0.8	0.79	1.07	**0.74**	**1.58**	3.09	1.81	**0.29**	0.35	0.37
1LC6	U6 stem loop	25		3.6	1.0	**0.31**	0.45	0.32	1.06	2.02	**1.01**	**0.25**	0.26	0.39
1KPY	PEMV-1 P1-P2 frameshifting pseudoknot	29	P	2.6	1.4	0.29	0.78	**0.28**	1.04	1.82	**0.98**	**0.53**	0.59	0.90
2LK3	Yeast U2/U2 snRNA complex	25		1.2	0.5	0.38	0.41	**0.32**	**1.27**	2.70	1.64	**0.30**	0.30	0.34
2JTP	Frameshift-inducing stem-loop in SIV	35		3.2	1.0	0.32	0.29	**0.25**	**1.29**	3.30	1.31	**0.37**	0.44	0.52
1PJY	HIV-1 frameshift inducing stem-loop	23		3.0	1.0	0.40	0.72	**0.32**	**1.22**	1.98	1.26	0.26	**0.22**	0.33
2LP9	Pseudo-triloop from BMV	17		1.8	0.8	0.47	1.33	**0.47**	1.24	2.19	**1.16**	**0.31**	0.33	0.55
1HWQ	VS ribozyme substrate	31		9.0	2.6	0.49	0.89	**0.41**	**0.56**	1.42	0.57	**0.37**	0.40	0.45
2L94	HIV-1 frameshift site bound to inhibitor	46		2.8	0.9	0.27	0.24	**0.20**	0.63	0.85	**0.56**	**0.28**	0.30	0.32
2L8H	HIV-1 TAR bound to probe	30		1.5	0.5	0.53	0.76	0.70	1.49	2.15	**1.40**	**0.32**	0.36	0.40
2QH4	scaRNA 5' term hairpin from human telom.	19		1.8	0.6	0.76	1.49	**0.67**	**1.24**	3.02	1.45	**0.30**	0.36	0.37
2LDL	HIV-1 exon splicing silencer 3	28		0.9	0.4	**0.25**	0.36	0.27	0.79	1.29	**0.70**	**0.18**	0.21	0.21
1R2P	D5 from ai5γ group II intron	35		6.3	2.3	0.43	0.62	**0.36**	0.53	1.00	**0.44**	0.34	**0.31**	0.38
2K63	EBS1 of group II intron Sc.ai5γ	30		4.1	2.0	**0.86**	1.15	1.20	1.23	1.99	**1.14**	**0.36**	0.39	0.44

PDB-id	Description	Length	Topology	Exploration radius	Avg. min $RMSD_{NMR}$	Helix $D_{KL}(M,KGSrna)$	Helix $D_{KL}(M,init)$	Helix $D_{KL}(M,NMR)$	Non-helix $D_{KL}(M,KGSrna)$	Non-helix $D_{KL}(M,init)$	Non-helix $D_{KL}(M,NMR)$	min $RMSD_{CS}(M,KGSrna)$	min $RMSD_{CS}(M,NMR)$	$RMSD_{CS}(M,init)$
2M58	2'-5' AG1 lariat forming ribozyme	59	P/B	22.9	9.8	0.62	0.80	**0.51**	0.50	0.64	**0.48**	**0.44**	0.51	0.55
2L6I	Coronoviral stemloop 2	17		0.9	0.3	0.40	0.47	**0.29**	**1.94**	2.64	2.46	**0.18**	0.19	0.22
4A4R	UAAC tetraloop	22		3.1	0.8	0.86	1.20	**0.75**	**1.19**	2.43	1.71	0.33	**0.33**	0.36
1BN0	SL3 hairpin	21		3.7	0.9	0.59	0.81	**0.53**	1.26	2.48	**1.13**	**0.23**	0.25	0.37
2L1V	PreQ1 riboswitch bound to preQ1	37	P	1.2	0.6	**0.74**	1.18	**0.74**	0.48	0.47	**0.35**	**0.48**	0.56	0.59
2LQZ	RNA claw of DNA packaging motor b29	28		2.1	1.0	0.65	1.13	**0.51**	**0.70**	1.23	0.89	0.35	**0.35**	0.47
2M4Q	Ribosomela decoding site of e.coli	28		6.0	1.7	0.62	0.79	**0.56**	0.66	1.19	**0.51**	0.39	**0.38**	0.44
2M21	Stem IV loop of Tetrahymena telomerase	22		2.1	0.8	**0.55**	0.88	0.59	1.36	2.21	**1.11**	0.31	**0.30**	0.42
2O32	U2 snRNA stem I from human	20		3.4	1.3	**1.50**	2.74	2.13	1.06	1.34	**0.75**	**0.35**	0.36	0.36
Min		15		0.7	0.3	0.20	0.24	0.20	0.24	0.35	0.27	0.16	0.17	0.20
Max		59		22.9	9.8	1.50	2.74	2.13	1.94	3.30	2.46	0.53	0.59	0.90
Avg		30		3.4	1.2	0.53	0.83	0.58	0.89	1.54	0.99	0.30	0.33	0.38

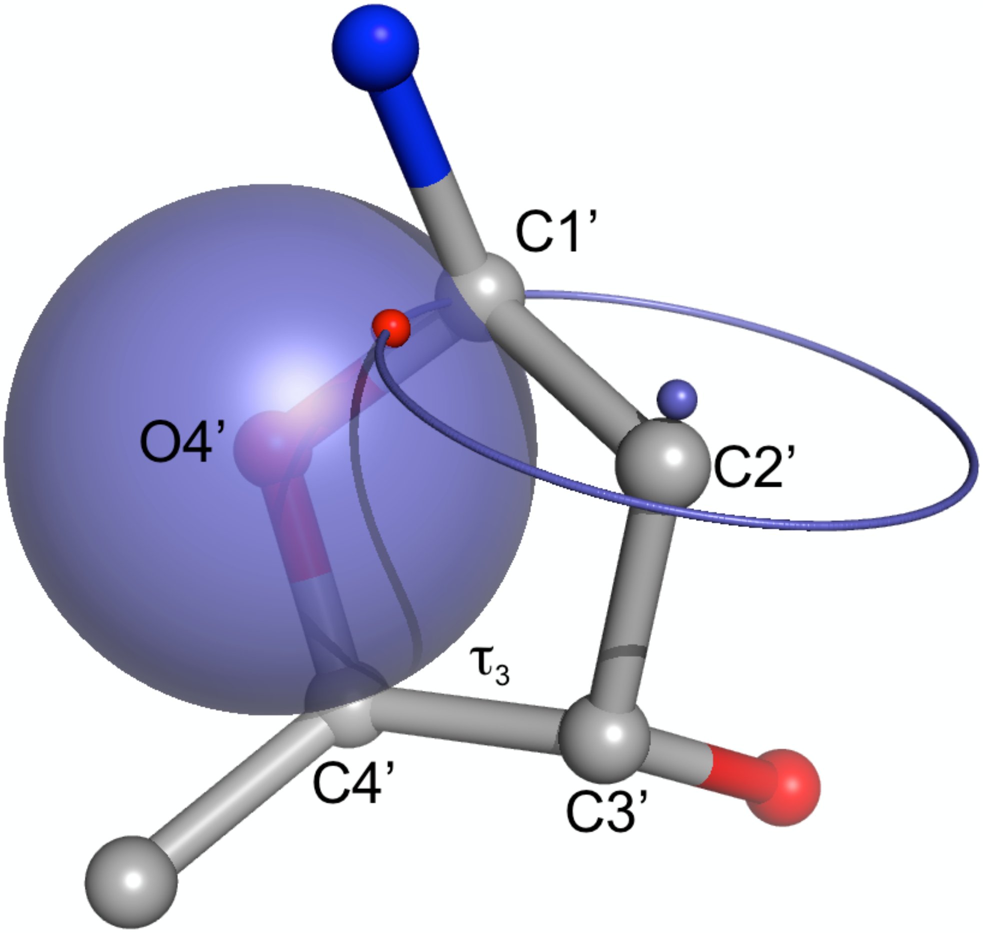

Supplementary Figure 1 Ribose puckering method. Perturbing backbone degrees-of-freedom generally breaks the geometry of riboses. We introduced a differentiable coordinate transformation to maintain ideal geometry of the ribose when the δ torsional degree-of-freedom is perturbed. The positions of O4', C4', and C3' are determined by (torsional) degrees-of-freedom earlier in the kinematic linkage. The position of C2' and the part of the kinematic linkage beyond C2' and C3' is determined from the O4'-C4'-C3'-C2' torsion, τ_3. Thus, the remaining atom C1' needs to be placed to maintain ideal geometry for the ribose. For a given value of τ_3, the position of C1' is determined by the intersection of a circle centered on the C2'-C3' axis, tracing positions of C1' with ideal C1'-C2'-C3' angle and C1'-C2' distance, and a circle centered on O4' tracing positions of C1' with ideal C1'-O4'-C4' angle and C1'-O4' distance. To allow for slightly non-ideal geometry, we replaced the latter circle with a spherical section. The position of C1' then must be on an intersection between a sphere and a circle. A binary variable, u, indicates which of the two circle-sphere intersections is the selected position of C1'. To avoid using the discontinuous variable u directly as an internal coordinate we introduce the periodic and continuous variable τ, which uniquely specifies both τ_3 and u. Since τ_3 is restricted to move in the range $[-A, A)$ where A is typically 41°, we set $\tau_3 = A \cos \tau$. By defining $u = sgn(\sin \tau)$, the ribose puckering follows a continuous, differentiable, and periodic motion for τ in $[0, 2\pi)$. The backbone C5'-C4'-C3'-O3' torsion, δ, is related to the ribose puckering by the relation $\delta = B + \tau_3$, where B is usually 120°.

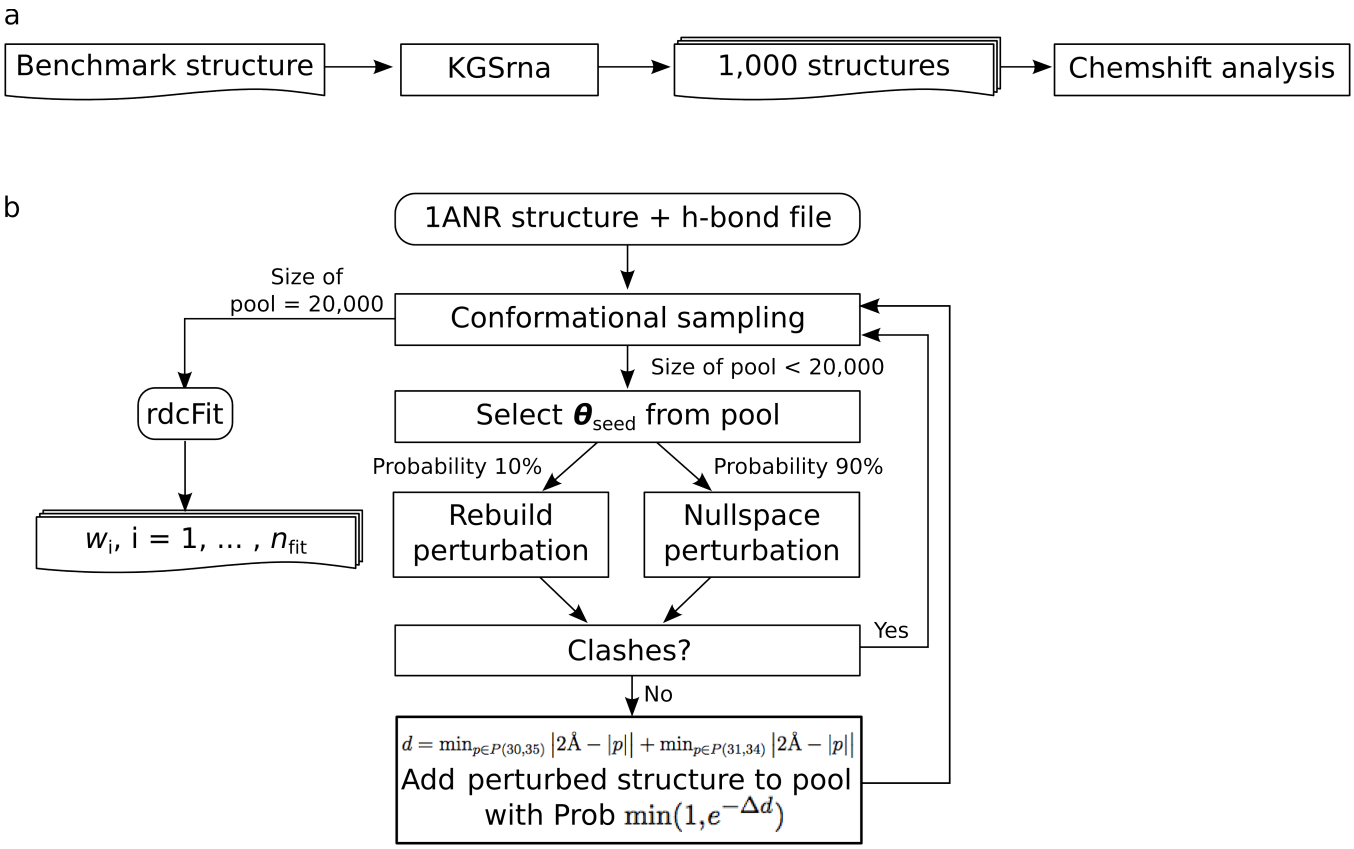

Supplementary Figure 2. Flowcharts of the KGSrna sampling algorithm used with experimental data. (**a**) For each of the 60 benchmark structures, 1,000 samples were generated with KGSrna. Chemical shifts were back-calculated from the 1,000 structures with the NUCHEMICS software. (**b**) We calculated 20,000 samples each starting from models one to ten in the NMR bundle of wild type HIV-1 TAR with PDB id 1ANR. The KGSrna sampling procedure was biased towards generically pairing the C30 and A35 bases and the U31 and G34 bases using a Metropolis criterion. The seed was selected from the pool based on Δd from the starting model. The procedure was repeated until 20,000 conformations were obtained. RDCs were back-calculated from the 20,000 structures with the PALES software.

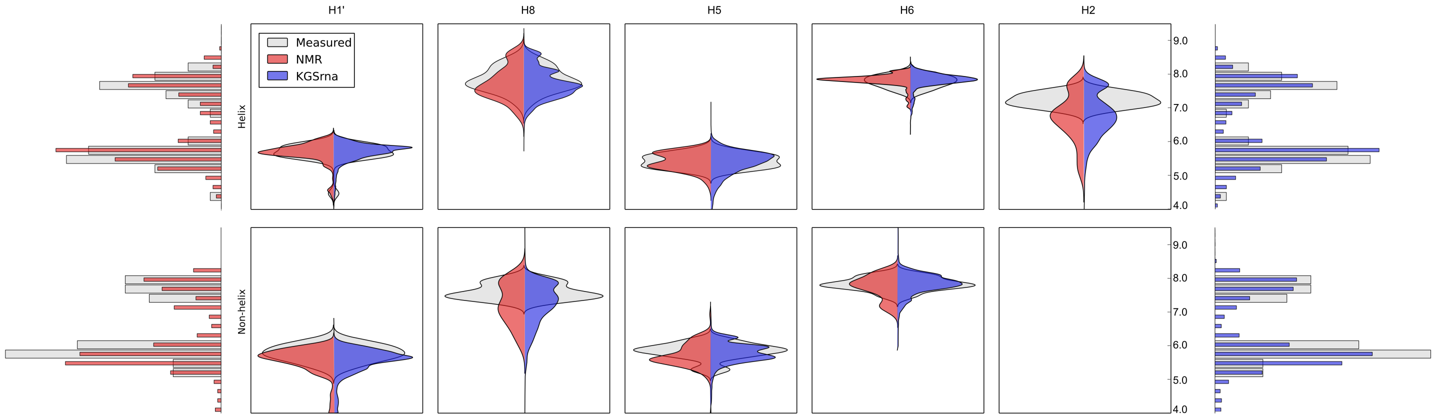

Supplementary Figure 3. Distributions of ^{1}H chemical shifts for P2a-J2a/b-P2b of human telomerase (PDB id 2L3E). P2a-J2a/b-P2b of human telomerase exhibits close to average sampling properties of the KGSrna ensemble, with an average minimum RMSD of 1.1Å to the NMR bundle. The top panel shows measured (grey), NMR bundle (red) and KGSrna ensemble (blue) ^{1}H chemical shifts. The discrete distributions were smoothed with a Gaussian Kernel Density Estimator for easier visualization. The superior agreement of the KGSrna distribution with the measured distribution compared to that of the NMR bundle is especially evident in non-helical regions. The symmetrized Kullback-Leibler divergence $D_{\mathrm{KL}}(\mathrm{P}^{\mathrm{M}} \,||\, \mathrm{P}^{\mathrm{KGSrna}})$ for helices is calculated from the upper right histogram as

$$\frac{1}{2}\sum_{i} P^{KGSrna}(i)\cdot\ln\left(\frac{P^{KGSrna}(i)}{P^{M}(i)}\right)+P^{M}(i)\cdot\ln\left(\frac{P^{M}(i)}{P^{KGSrna}(i)}\right),$$

where $P^{\bullet}(i)$ is the number of observations in bin *i*. For helices in 2L3E, $D_{\mathrm{KL}}(\mathrm{P}^{\mathrm{M}} \,||\, \mathrm{P}^{\mathrm{KGSrna}})$ = 0.17 bits whereas $D_{\mathrm{KL}}(\mathrm{P}^{\mathrm{M}} \,||\, \mathrm{P}^{\mathrm{NMR}})$ = 0.32 bits. This is commonly interpreted as an information gain of 0.15 bits when using the KGSrna distribution instead of the NMR distribution to approximate the true distribution. Likewise, for non-helical regions $D_{\mathrm{KL}}(\mathrm{P}^{\mathrm{M}} \,||\, \mathrm{P}^{\mathrm{KGSrna}})$ = 0.27 bits and $D_{\mathrm{KL}}(\mathrm{P}^{\mathrm{M}} \,||\, \mathrm{P}^{\mathrm{NMR}})$ = 0.58 bits, an information gain of 0.31 bits.

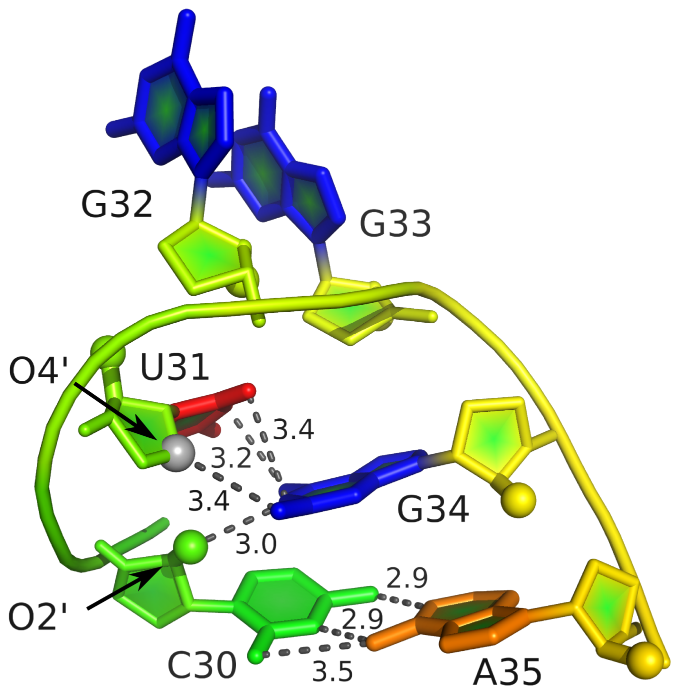

Supplementary Figure 4. A snapshot along the molecular dynamics trajectory structurally characterizing the ES of the HIV1-TAR apical loop. The non-canonical closing base-pair of the HIV1-TAR apical loop forms stable hydrogen bonds C30(N4)—A35(N1) and C30(N3)—A35(N6), both with a length of 2.9Å, and a weaker C30(O2)—A35(N6) (3.5Å). The C30 glycosidic angle is *anti* (-143.9°), and its ribose conformation is C2'-endo. A35 adopts a *syn* base (44.2°) with a C3'-endo ribose conformation. G34 is tucked deeply into the loop to adopt a wobble pair with U31 through hydrogen bonds U31(O2)—G34(N1) (3.4Å) and U31(N3)—G34(O6) (3.2Å). U31 (*anti*) and G34 (*anti*) are not co-planar, but U31 is staggered towards the apex of the loop and slightly looped out. G34 is further stabilized by interactions with the U31 ribose (O4', grey sphere), and the C30 hydroxyl group.

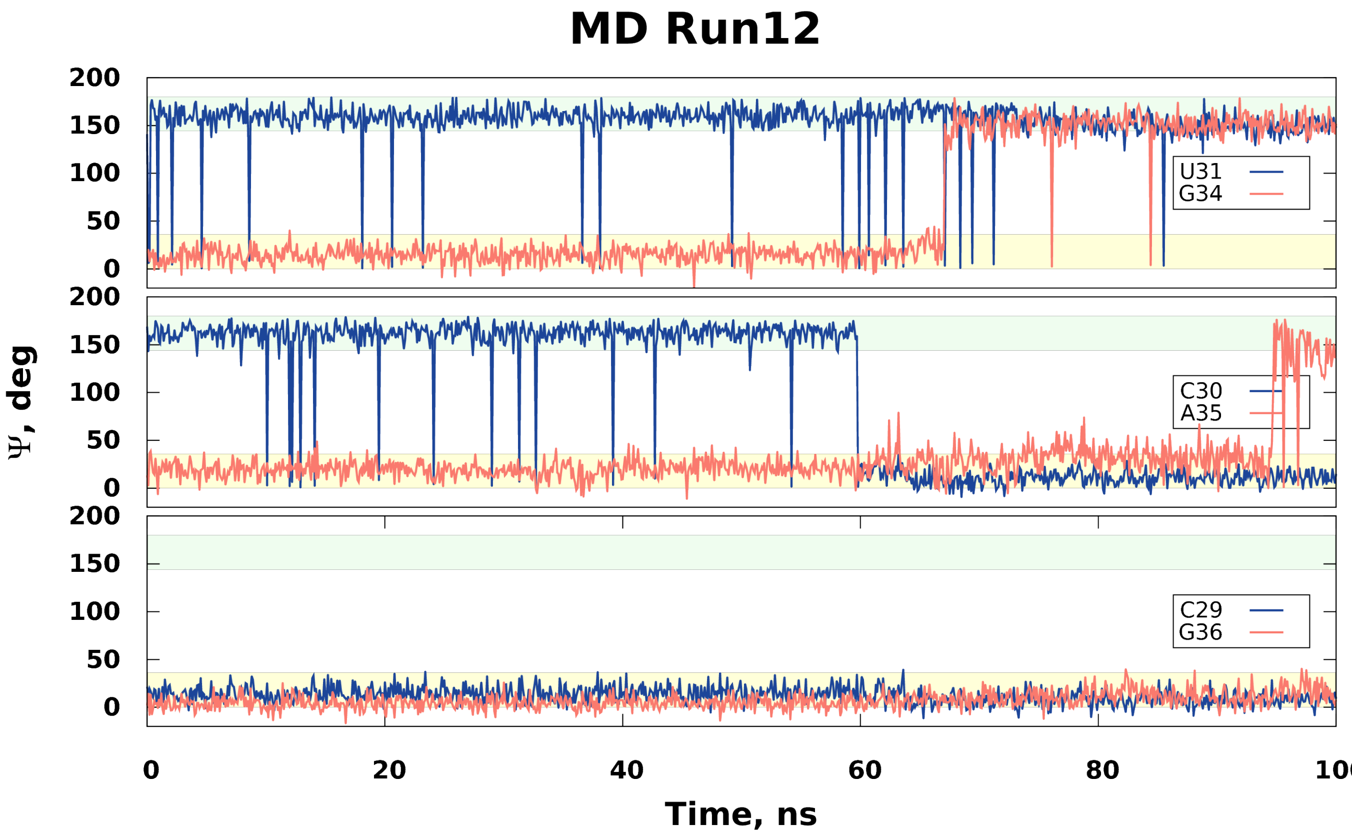

Supplementary Figure 5. Time evolution of ribose puckers for HIV1-TAR apical loop nucleotides. The panels show the puckering angle for riboses of paired nucleotides in the HIV1-TAR apical loop for the duration of the MD simulation. While paired, sugar puckers are either C3'-endo (lower bands) or C2'-endo (upper bands) and continuing the helical stem.
